# Functional assertiveness with acceptance and commitment therapy for employees returning to work: a preliminary investigation

**DOI:** 10.3389/fpsyg.2025.1415212

**Published:** 2025-01-24

**Authors:** Daisuke Ito, Yushun Okabe, Aya Nobushige, Seiko Saito, Yoshinari Takahashi

**Affiliations:** ^1^Graduate School of Education, Hyogo University of Teacher Education, Kato, Japan; ^2^Tanimachi Central Stress Care Clinic, Osaka, Japan; ^3^Joint Graduate School (Ph.D. Program) in Science of School Education, Hyogo University of Teacher Education, Kato, Japan; ^4^Japan Society for the Promotion of Science, Tokyo, Japan; ^5^LITALICO Partners Inc., Tokyo, Japan; ^6^Utsumi Mental Clinic, Nishinomiya, Japan

**Keywords:** functional assertiveness, rework program, acceptance and commitment therapy, pilot study, preliminary investigation

## Abstract

Owing to the ongoing global mental health crisis, providing support for employees returning to work after a mental health leave has become a crucial issue. This study aimed to preliminarily examine the efficacy of a functional assertiveness training program incorporating acceptance and commitment therapy (ACT) in a pilot study involving individuals who had taken a leave of absence due to mental health problems. As part of the program, eight group sessions were offered to these individuals in a group format while they attended an outpatient psychiatric clinic. The effectiveness of the program was assessed in terms of mindfulness, psychological flexibility, functional assertiveness, and difficulty in returning to work. A total of 29 participants took part in the program, and 28 participants completed it. A paired *t*-test and effect size analysis (Cohen’s *d*) were conducted using data from 26 participants; two participants were excluded from the analysis due to post-test data was not collected. The results showed an increase in participants’ mindfulness (observing, non-reactivity and non-judging), psychological flexibility, and functional assertiveness increased and their sense of difficulty in returning to work (especially difficulties arising from workplace relationships) decreased after the intervention. Although the lack of a control group makes it difficult to draw definitive conclusions, the results suggest that a functional assertiveness training program incorporating ACT may be beneficial for employees on leave due to mental health issues in returning to work.

## 1 Introduction

Many companies face an increase in employee absences due to mental health issues. In Japan, 82.2% of workers reported experiencing significant anxiety, worry, and stress related to work and professional life (Ministry of Health, Labor and Welfare, 2023). Additionally, 13.5% of establishments had employees who took leaves of absence for more than a month or resigned because of mental health problems in the past year, and this percentage is on the rise (Ministry of Health, Labor and Welfare, 2023). Consequently, companies are attempting to facilitate these employees’ return to work (RTW), and guidelines have been developed (Igarashi, 2018). In particular, cognitive behavioral therapy (CBT) is often used in this process, as it has been shown to improve mental health (Tanoue et al., 2012). By helping individuals develop cognitive and coping strategies for daily stress, CBT can reduce depression and anxiety, thereby improving chances for RTW (Watanabe et al., 2019). A large-scale randomized controlled trial found that work-focused CBT improves health-related quality of life and workplace participation, with those undergoing work-focused CBT returning to work quicker than those receiving typical care (Xu et al., 2024).

Recent research has emphasized the importance of re-examining psychological support for RTW (Watanabe et al., 2019). This support framework prioritizes the enhancement of retention rates after RTW and the prevention of relapses rather than solely focusing on the facilitation of RTW (Endo et al., 2019). This is because, although traditional CBT focuses on correcting maladaptive thoughts and cognitive distortions, even when cognitive modifications appear successful, the thought patterns can be reactivated in the workplace after RTW, potentially leading to relapse (Tanoue et al., 2012). Consequently, in recent years, acceptance and commitment therapy (ACT) has gained attention for improving the mental health of individuals on leave. This therapy focuses on the relationship between individuals and their thoughts and feelings rather than the content of the cognition (e.g., Klevanger et al., 2018). The ACT-based RTW rehabilitation programs for workers on leave due to chronic fatigue have been investigated in several countries (Brugnera et al., 2021), with the results showing the long-term cost-effectiveness of these programs for individuals on leave due to mental disorders (Finnes et al., 2022). However, no studies have been conducted to examine the use of ACT to support people returning to work in Japan.

Among the 687 Japanese companies reporting cases of workers being absent or taking leave due to mental health issues in the past year, the most common reason was human relations in the workplace (323 companies or 47%; Osaka Labor Bureau, 2011). In Japan, the principle of “returning to the current position” is often adopted to minimize the burden of adjusting to a new job and workplace (Igarashi, 2018). Thus, employees on leave return to their former workplaces where relationships were problematic. Nevertheless, many of those on leave have strong concerns about building interpersonal relationships in the workplace, hindering their RTW and workplace retention (Watanabe et al., 2019). Indeed, problems related to interpersonal relationships in the workplace can impact employment stability after RTW (Nilsen et al., 2023). Therefore, it is essential for people on leave to acquire skills that help build good relationships with superiors and colleagues in the workplace (Igarashi, 2018).

Behavioral approaches for promoting strong interpersonal relationships include social skills and assertiveness training, which are applied in the education and welfare fields (e.g., Spence, 2003). These approaches focus on learning socially desirable standard behaviors and clearly expressing opinions and demands. Assertiveness training has become more popular than behavioral therapy in Japan as a comprehensive program based on the belief that people have the right to assert themselves. Consequently, there is a strong emphasis on the idea that assertiveness is valid. However, research has suggested that this logical and straightforward assertiveness may be perceived as aggressive and hostile (Wilson and Gallois, 1998). In particular, since Japanese culture tends to prioritize group harmony over individual opinions (Markus and Kitamura, 1991), straightforward expression may easily lead to conflicts in interpersonal relationships. Additionally, the situational context should be considered (Mitamura, 2018). For example, in the workplace, where interactions with superiors, coworkers, and customers of various backgrounds and positions are expected, even if a certain behavior is generally considered to be acceptable, it may be judged as inappropriate depending on the context. Therefore, for assertiveness to be functional and practical in the workplace, it is essential to respect one’s own thoughts and feelings while fully considering the workplace context (Nobushige et al., 2023). This involves continually devising ways to facilitate effective communication (Mitamura, 2018).

To address the aforementioned issues and enhance assertiveness techniques, Mitamura and Tanaka-Matsumi (2009) introduced the concept of “functional assertiveness,” which integrates both objective effectiveness (the speaker achieves the desired effect as a result of self-expression) and practical politeness (the listener perceives the speaker’s expression as situationally appropriate). Functional assertiveness involves interpersonal communication occurring when a speaker presents an interpersonal problem requiring resolution or an objective that must be achieved, and the listener perceives the speaker’s message as situationally appropriate (Mitamura and Tanaka-Matsumi, 2010). In this communication, the speaker and listener understand each other’s perspectives and are sensitive to the social context, which determines the acceptability of assertiveness. Mitamura and Tanaka (2014) developed a functional assertiveness training program for parents who wanted to ask elementary school teachers to support their children with developmental disabilities more effectively (in Japan, it is a very delicate situation for parents to ask for demands from schoolteachers). This pilot study suggested that parents’ functional assertiveness improved. In other words, after the intervention, teachers evaluated the way in which parents expressed their requests as more polite, specific, and acceptable than before the intervention. However, this program focused on acquiring skills through role-playing and other methods so that parents could express their appreciation and consideration to teachers while embodying the content of their request. Hence, incorporating other aspects into the program could enhance sensitivity to the social context, a key feature of functional assertiveness. In other words, functional assertiveness involves not only learning communication skills but also developing a strategy to understand one’s desired outcomes and consider the social context to express them effectively, but previous programs did not include procedures for achieving this.

Shigemoto (2021) proposes the adoption of ACT concepts and techniques to enhance functional assertiveness. ACT is based on functional contextualism and aims to increase psychological flexibility and has been shown to be more effective for interpersonal problems than other psychotherapies (Blume and Keng, 2018). Functional assertiveness aims to achieve the effect the speaker expects by selecting an expression method according to the context (e.g., relationship with listener and situation of assertion), regardless of the form of the assertiveness behavior. This is consistent with the functional contextualism of ACT, which focuses on understanding the effectiveness of an action in the context in which it is currently being performed. In addition, Shigemoto (2021) states that reading the social context and adopting the perspective of others is essential for making functional assertions and acquiring this perspective is one of the goals of ACT. These are skills that are difficult to acquire through language instruction alone and have not been adequately addressed in previous functional assertiveness programs. In this regard, ACT is particularly sensitive to the context, function, and form of psychological phenomena and has a wealth of change strategies based on context and experience besides more direct guidance such as role play (Hayes et al., 2003). In other words, utilizing ACT would be useful in functional assertiveness programs as it has specific methods for improving the abilities needed for functional assertiveness (e.g., awareness of context, clarification of personality chosen behavior, values).

Furthermore, ACT can also be expected to promote acceptance of difficult thoughts and feelings and encourage actions aligned with meaningful and valued living, even in the presence of challenging circumstances (Hayes et al., 2003). For example, in the workplace, thoughts and feelings, such as “fear of negative feedback from supervisors or customers” or “impatience to work to the best of my ability as soon as I return to work to make up for lost time,” can lead to losing sight of the original goals and values of the job and oneself, resulting in ineffectual contextual communication. By embracing ACT, individuals can acknowledge and accept these challenging thoughts and feelings, assess reality as it is, and set goals aligned with their core values (the direction they deem important in life). This approach can contribute to improving functional assertiveness because it promotes flexible communication in the workplace and allows each individual to maintain their own values. This clarification of values through ACT is expected to enhance the effectiveness of functional assertion. This is because functional assertion has two aspects: achieving one’s goals and being considerate of others; however, these two aspects can cause conflict situations. By recognizing the importance of being considerate of one’s own and of others’ values, it becomes possible to choose to act in a way that addresses both aspects. For example, if you value your colleague’s feelings, you may need to turn down a request from them to get something more important to you such as family time. In such situations, you may recognize that your family is a high priority for you. By asserting yourself and communicating your values to the other person, you will be able meet your needs while still being considerate to your colleague. Ultimately, this will lead to the realization of functional assertiveness.

Based on the above ideas, Nobushige et al. (2023) developed a functional assertiveness training program incorporating ACT tailored for individuals on leave from work. When implemented in a single-case study, the recipient experienced a reduced sense of difficulty in RTW. However, owing to the single-case study design, we were unable to explore any changes in the process with statistical confidence (e.g., functional assertiveness and ACT components such as mindfulness and psychological flexibility) or outcome variables (e.g., difficulty in RTW) and generalize the findings. Therefore, this study aimed to preliminarily examine the efficacy of a functional assertiveness training program incorporating ACT in a pilot study involving individuals who had taken a leave of absence due to mental health problems. The program’s effectiveness was evaluated by examining whether the outcome variable improved after the program. Previous functional assertiveness programs did not include enough intervention elements to improve sensitivity to social contexts (e.g., Mitamura and Tanaka, 2014). Hence, if the effectiveness of a program that includes ACT elements can be verified, ACT can be established as one method for improving the effectiveness of functional assertiveness, thus enriching basic research on functional assertiveness and the expansion of support for RTW.

## 2 Methods

### 2.1 Participants

The participants were outpatients of a mental health clinic offering general medical services who were on leave due to psychological symptoms and hoped to RTW. The eligibility criteria were (1) adults aged ≥20 years, (2) those whose attending physician determined that their mental health issues had resulted in a leave of absence, (3) those who were currently on leave but wished to RTW, and (4) those with a referral from their attending physician to participate in this study. Exclusion criteria included (1) current or previous diagnosis of a psychotic spectrum disorder or evidence of an organic brain disorder, developmental delay, or personality disorder; (2) current high risk of suicide; (3) substance abuse; and/or (4) major somatic disease. Participants were recruited by psychiatrists who reviewed the eligibility and exclusion criteria and identified study participants’ diagnoses. All participants provided informed consent, and all research procedures were conducted with the approval of the Research Ethics Committee affiliated with the first author.

**Figure 1:**
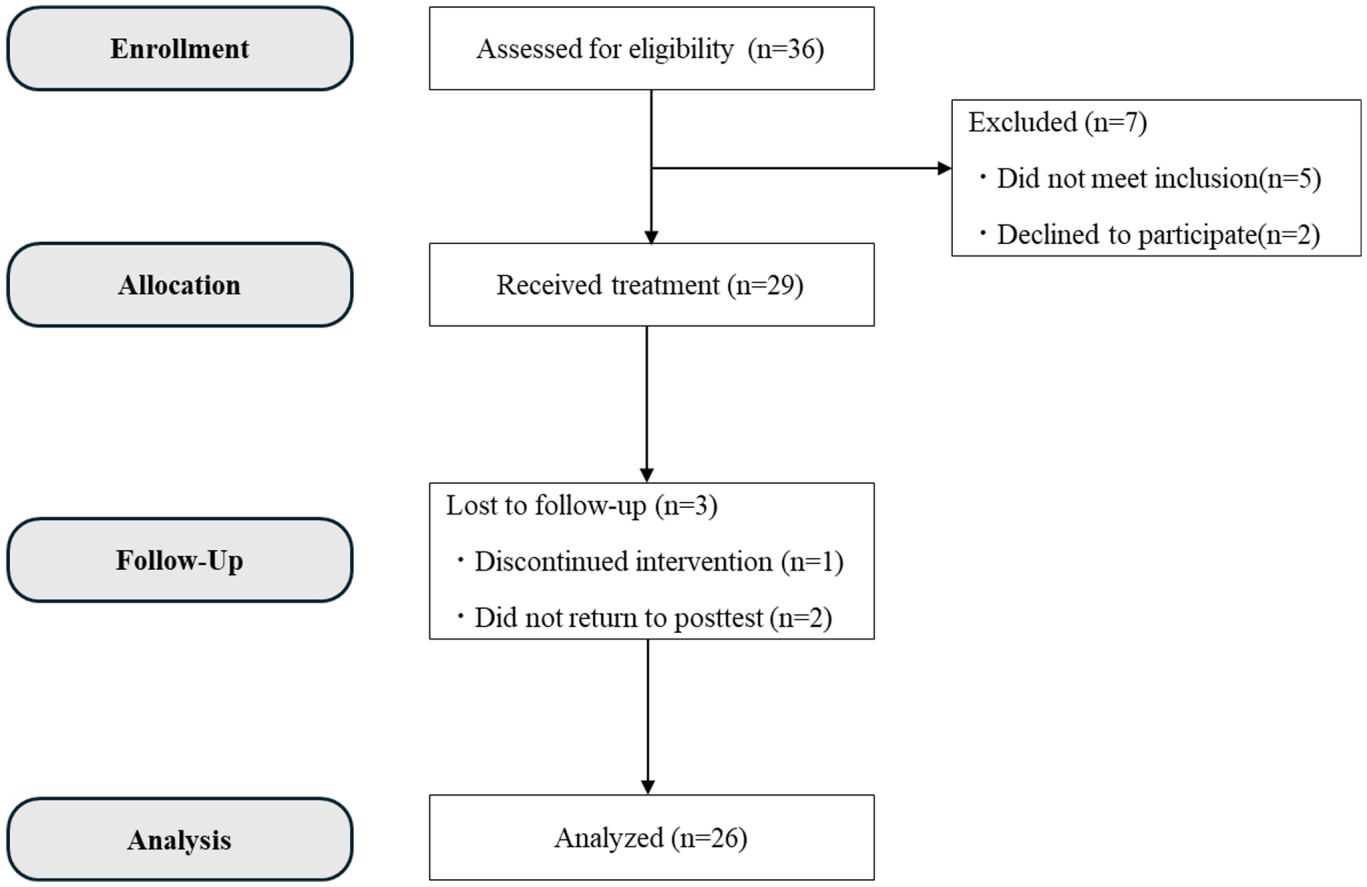
CONSORT flowchart of participants’ sampling procedure.

An a priori power analysis (*ES* = 0.5, *α* = 0.05, *β* = 0.80) revealed that at least 36 participants were necessary. Figure 1 presents the participants’ sampling procedure. Based on the screening criteria, 36 patients were screened. Of these, 29 participated in the program, but one dropped out halfway through, so 28 participants completed the program (a dropout rate of 3.45%). Additionally, two more were excluded from the analysis because they had incomplete responses to the post-test, so the final number of participants analyzed was 26 participants (16 men, 10 women; M_age_ = 37.81 years, standard deviation = 9.74 years) who successfully completed the intervention and were included in the analysis. Their diagnoses were as follows: adjustment disorder with depression and anxiety (*n* = 20), major depressive disorder (single episode; *n* = 10), major depressive disorder (recurrent episodes; *n* = 4), autism spectrum disorder (*n* = 2), persistent depressive disorder (*n* = 1), and adjustment disorder with anxiety (*n* = 1). It should be noted the 16 participants (61.53%) had comorbid mental health conditions.

### 2.2 Treatment procedure

The program involved eight 60-min sessions for groups of 2–4 patients conducted by a psychologist who had been studying ACT for over 5 years and a nurse who had been studying RTW support for over 10 years. The intervention was not conducted simultaneously for all participants, but for a total of nine groups. Session 1 involved recognizing one’s communication tendencies. In Sessions 2–4, explanations on ACT necessary for effective implementation of functional assertiveness were given, including acceptance techniques and clarification of values, in addition to conveying the methods of expression that are considerate to the other party. Sessions 5–8 involved roleplaying using functional assertiveness in different workplace conflict situations. During the program, participants were assigned homework to ensure that they practiced mindfulness on a daily basis. After each session, the attending physician and intervention staff met to discuss and share information about the patient’s performance according to the treatment plan and their health status. The attending physician, who had over 10 years of practicing ACT and had specialized knowledge of functional assertion, supervised to ensure that the program was being implemented in accordance with the program manual. Table 1 summarizes the timeline and contents of the treatment procedure (Perera et al., 2007).

**Table 1:** Intervention contents based on time and contents of the functional assertiveness training program.

Timeline	Functional assertiveness training program	Regular treatment
Interview for program		a, b, c
Pre-intervention	Baseline assessment (pre)	
Intervention (2 months)	d, e	c
Post-intervention	Post assessment (post)	
*1. Recognize your communication tendencies* •Lecture: (1) Overall program description. (2) Introduction to functional assertiveness •Exercise: A worksheet to identify communication tendencies in various areas of life (e.g., workplace, family, friends, and significant others). *2. Psycho-education on functional assertiveness with the aid of ACT* •Lecture: (1) Learning the importance of expressing opinions while considering the context of human relationships and being considerate of others. (2) Learning the importance of being present with inner experiences without being attached to them using acceptance techniques. •Exercise: (1) Practice acceptance techniques (mindfulness and defusion) and learn how to observe one’s experiences without being caught up in thoughts and feelings. For example, practice exercises such as “Leaves on a Stream” and “Labeling your Thoughts” (Harris, 2012). •Homework (all sessions after the second): Practice mindfulness at home. However, they were encouraged to report on their progress and any questions they had as situationally appropriate. *3. Clarify your own values* •Lecture: Explain the importance of clarifying the value of ACT in assertions. •Exercise: Identify values in different areas of life through discussions with other patients. For example, practice exercises such as “The Life Compass” and “Value Cards” (Harris, 2012) *4. Understand how to practice functional assertiveness with the aid of ACT* •Lecture: (1) Learn how to express yourself with consideration for others. (2) Explain the importance of using them in a social context. (3) Explain that negative thoughts and unclear values make it difficult to grasp the context. •Exercise: Imagine a difficult communication situation in the workplace. Participants will discuss the difference between the traditional approach and the functional assertiveness-based approach with the help of ACT when dealing with such a situation. *5–8. Practice of functional assertiveness with the aid of ACT* •Exercise: (1) Practice communicating in a way that is consistent with one’s values while accepting problematic inner experiences. (2) Roleplay making a request, consulting, expressing opinions, and reporting mistakes in the workplace. (3) Modify communication behavior based on the results of role-play practice.		

ACT, acceptance and commitment therapy. ^a^Comprehensive history interview. ^b^Comprehensive assessments from biological, psychological, and social perspectives. ^c^Regular medical care is provided by the attending physician. There are no restrictions on medication, other psychological interventions, or referrals to specialized agencies. ^d^Materials for the program (e.g., worksheets and homework), which consists of eight sessions (one per week), are handed to the participants in each session. Homework is assigned so that the participants implement mindfulness training every day. ^e^A functional assertiveness training program, consisting of eight 60-min sessions, is conducted by one psychologist and one nurse for each group of 2–4 participants. After each session, the attending physician and intervention staff hold a meeting to discuss the patient’s performance according to the treatment plan and the participant’s health status.

### 2.3 Variables and measures

The process variables were mindfulness, psychological flexibility, and functional assertiveness, and the outcome variable was difficulty in RTW. Evaluation was carried out using a questionnaire at two time points: before the program began and 2 months after its completion.

#### 2.3.1 Demographic data

We asked for information on age and gender.

#### 2.3.2 Mindfulness

Participants’ mindfulness was measured using the Japanese version of the Five Facet Mindfulness Questionnaire (FFMQ) developed by Sugiura et al. (2012) and introduced by Baer et al. (2006). The Japanese version comprises 39 items, with scores for each item ranging from 1 = *Not at all* to 5 = *Always*. The FFMQ has five subscales due to (1) Observing (Paying attention to internal and external experiences, such as thoughts, emotions, and sensations), (2) Non-reactivity (Allowing thoughts and feelings to come and go without getting caught up in them), (3) Non-judging (Taking a non-evaluative stance toward thoughts and feelings), (4) Describing (Labeling internal experiences with words), (5) Acting with awareness (Focusing on what is happening in the moment, instead of acting on automatic pilot). Higher scores suggest greater level of mindfulness. Sugiura et al. (2012) demonstrated its reliability and validity. Since mindfulness was practiced outside the program as homework in this study, the FFMQ, which can evaluate mindfulness in various ways, was used. The reliability of this study was calculated (*α* = 0.81).

#### 2.3.3 Psychological flexibility

Participants’ psychological flexibility was measured using the Japanese version of the Acceptance and Action Questionnaire II (AAQ-II) developed by Shima et al. (2013), introduced by Bond et al. (2011). The Japanese version includes seven items, with scores for each ranging from 1 = *Not at all* to 7 = *Always*. Lower scores suggest higher levels of psychological flexibility. Shima et al. (2013) demonstrated its reliability and validity. The reliability of this study was calculated (*α* = 0.80).

#### 2.3.4 Functional assertiveness

Participants’ functional assertiveness was measured using the original Functional Assertiveness Scale (FAS) developed by Mitamura (2018). This 12-item self-report scale has scores for each item ranging from 1 = *Always disagree* to 5 = *Always agree*. The FAS assesses individuals’ satisfaction with the outcomes in certain situations, such as wanting someone to do or stop doing something. In this study, using the FAS, we evaluated whether participants could effectively manage interpersonal conflicts and problems contextually. The FAS has two subscales. The first subscale, objective effectiveness, measures whether individuals can effectively handle interpersonal conflicts or problems themselves. An example item is, “I can get people to understand my ideas even if they are different from theirs.” The second subscale, pragmatic politeness, measures whether individuals can effectively handle interpersonal conflicts or problems from others’ perspectives. An example item is, “I do not get on a person’s nerves more than necessary when I try to get them to understand that they are being unjust in pointing out my failures.” Higher scores suggest greater level of functional assertiveness. Mitamura (2018) demonstrated FAS’s reliability and validity. The reliability of this study was calculated (*α* = 0.85).

#### 2.3.5 Difficulty in RTW

Difficulty in RTW was measured using the original Difficulty in Returning to Work Inventory (DRW) developed by Tanoue et al. (2012). This 10-item self-report scale has scores for each item ranging from 1 = *Not at all* to 4 = *Extremely difficult*. It was designed to assess the level of difficulty one experiences in RTW before participating in rework programs. The DRW has three subscales measuring difficulty in RTW due to (1) physical fitness (e.g., “I’m worried whether I have the physical strength required for when work time increases”), (2) human relationships (e.g., “I appear fine from the outside, so I’m worried whether co-workers will understand my sickness”), and (3) cognitive function (e.g., “I am worried whether I can concentrate efficiently on work”). This study specifically focused on the human relationships subscale. Higher scores suggest greater level of difficulty in RTW. Tanoue et al. (2012) demonstrated this scale’s high reliability and validity. The reliability of this study was calculated (*α* = 0.86).

### 2.4 Data analysis

Given the relatively small dataset, we conducted a Shapiro–Wilk test to check the normality assumptions for the FFMQ, AAQ-II, FAS, and DRW. We then conducted a paired *t*-test and Cohen’s *d* (Cohen, 1988) using the scores for each scale before and after the intervention to clarify the effectiveness of the program. We also conducted a post-hoc analysis of the mean difference between the two groups for the FAS score to confirm the test power. In general, a power of 0.8 or higher is considered appropriate (Cohen, 1988). All statistical analyses were performed using the IBM SPSS Statistics version 29 (IBM Corp., Armonk, NY).

## 3 Results

The results of the Shapiro–Wilk test showed that there was no significant difference, and that the normality of each scale could be assumed. After conducting paired *t*-tests, no significant difference was found in the scores for describing (*t*(25) = −1.50, *p* = 0.17, *d* = 0.24, 95% confidence interval [CI] [−0.10, 0.56]) and acting with awareness (*t*(25) = −1.46, *p* = 0.16, *d* = 0.20, 95% CI [−0.08, 0.47]), physical fitness (*t*(25) = 0.43, *p* = 0.67, *d* = 0.05, 95% CI [−0.18, 0.28]), cognitive function after the intervention (*t*(25) = 1.13, *p* = 0.27, *d* = 0.13, 95% CI [−0.11, 0.61]) (Table 2). However, scores for the overall FFMQ (*t*(25) = −3.32, *p < 0*.00, *d* = −0.63, 95% CI [0.20, 1.0]) and observing (*t*(25) = −4.12, *p < 0*.00, *d* = 0.61, 95% CI [0.28, 0.93]), non-reactivity (*t*(25) = −3.45, *p < 0*.00, *d* = 0.73, 95% CI [0.24, 1.2]), non-judging (*t*(25) = −2.80, *p = 0*.01, *d* = 0.45, 95% CI [0.10, 0.79]), AAQ-II (*t*(25) = 2.24, *p = 0*.03, *d* = 0.40, 95% CI [0.02, 0.78]), overall FAS (*t*(25) = −3.67, *p* < 0.00, *d* = −0.57, 95% CI [0.23, 0.92]), objective effectiveness (t(25) = −3.33, *p* < 0.00, *d* = −0.44, 95% CI [0.15, 1.1]), pragmatic politeness (*t*(25) = −2.71, *p* = 0.01, *d* = 38, 95% CI [0.08, 0.68]), overall DRW (*t*(25) = 3.11, *p < 0*.00, *d* = 0.31, 95% CI [0.10, 0.52]), human relationships (*t*(25) = 3.80, *p < 0*.00, *d* = 0.53, 95% CI [0.22, 0.84]) differed significantly after the intervention. Moreover, a post-hoc analysis was conducted on the FAS, the key measure in this study, and the power (1-*β*) was calculated as 0.80.

**Table 2:** Results of paired *t*-tests and determining the effect size.

	Pre-intervention		Post-intervention		*t*	*p*	*d*^a^	95% CI
	*M*	*SD*	*M*	*SD*				
FFMQ	111.19	20.05	122.88	16.14	−3.32	0.00	0.63	[0.20, 1.1]
Observing	24.42	4.82	27.31	4.71	−4.12	0.00	0.61	[0.28, 0.93]
Non-reactivity	17.46	4.26	20.50	4.10	−3.45	0.00	0.73	[0.24, 1.2]
Non-judging	21.92	7.26	25.04	6.62	−2.80	0.01	0.45	[0.10, 0.79]
Describing	22.65	6.45	24.08	5.34	−1.50	0.17	0.24	[−0.10, 0.56]
Acting with awareness	24.73	6.59	25.96	5.86	−1.46	0.16	0.20	[−0.08, 0.47]
AAQ-II	29.50	8.84	26.27	6.89	2.24	0.03	0.40	[0.02, 0.78]
Overall FAS	36.92	6.16	40.15	4.44	−3.67	0.00	0.57	[0.23, 0.92]
Objective effectiveness	15.85	4.71	17.81	3.53	−3.33	0.00	0.44	[0.15, 1.1]
Pragmatic politeness	21.08	3.54	22.35	2.38	−2.71	0.01	0.38	[0.08, 0.68]
Overall DRW	27.04	5.27	25.27	5.86	3.11	0.00	0.31	[0.10, 0.52]
Physical fitness	7.62	2.37	7.50	2.42	0.43	0.67	0.05	[−0.18, 0.28]
Human relationships	11.35	2.71	9.96	2.43	3.80	0.00	0.53	[0.22, 0.84]
Cognitive function	8.08	2.12	7.81	2.12	1.13	0.27	0.13	[−0.11, 0.61]

FFMQ, Five Facet Mindfulness Questionnaire; AAQ-II, Acceptance and Action Questionnaire-II; FAS, Functional Assertiveness Scale; DRW, Difficulty in Returning to Work Inventory; CI, confidence interval. ^a^Cohen’s d = 0.20 (small), 0.50 (medium), and 0.80 (large).

## 4 Discussion

The results suggest that participants’ mindfulness, psychological flexibility, and functional assertiveness increased after joining the program, while the difficulty of RTW due to interpersonal relationships was reduced. However, the scores for the two sub-factors of describing and acting with awareness in the FFMQ did not change. These factors include verbalizing one’s inner feelings and thoughts and acting intentionally while being aware of the “here and now.” However, in this study, the mindfulness intervention given as homework in the form of meditation done by the participants alone, so it is possible that the results were affected by the fact that the participants did not have the opportunity to verbalize or act in this way. In other words, one of the reasons for this finding may be that the program did not include mindfulness exercises such as the describing during the time it was implemented.

Although there are some variables that have not changed in this way, overall, it is suggested that incorporating mindfulness and value work elements of ACT may have increased awareness of social context and functional assertiveness. This can reduce the sense of difficulty for employees when returning to work. However, as this was a pilot study, it was not possible to confirm whether the effects of the program were actually achieved through this process, making it challenging to draw strong conclusions. Nonetheless, our results align with previous case studies suggesting the effectiveness of ACT with functional assertiveness for employees intending to RTW (Nobushige et al., 2023). This study contributes to the existing literature by including process and outcome variables and utilizing statistical methods. In addition, the results obtained support Shigemoto’s (2021) suggestion that ACT can be applied to functional assertiveness. Further, they indicate that conducting basic and clinical research from the perspective of ACT can contribute to developing functional assertiveness in the workplace.

Furthermore, it was possible that participants having frequent reminders of the workplace before returning to work, by participating in this program, could have been invasive and could have affected program acceptance. Igarashi (2018) reported a dropout rate of 22.8% for the RTW program. However, in this study, although the final number of participants analyzed was 26 out of 29, 28 of them completed the intervention (a dropout rate of 3.45%), suggesting that it was well accepted. Therapists using ACT for RTW indicated that identifying personal motivations for work provides a stronger foundation for securing work participation than the normative assumption that one must work (Klevanger et al., 2018), aligning with the values-based approach emphasized in this study. In other words, while it is difficult to establish a clear causal relationship, it is possible that the program’s focus on clarifying personal values related to work and addressing negative thoughts and feelings has resulted in participants continuing their involvement. This aspect will need to be investigated further in future studies.

Although literature recommends evidence-based interventions for employees on leave due to mental health problems, few studies have been conducted in Japan. It is unclear whether treatment methods that have been shown to be effective in Western countries can be also effective in Japan (Fujisawa et al., 2010). Hence, it is necessary to consider the issues in Japan, rather than simply using support methods that have been used in other countries. For example, in Japan, there is no established method that fully takes into account the context of the workplace, thereby highlighting the significance of developing an assertiveness program that can reduce the sense of difficulty in RTW in Japan. In addition, many RTW support programs in Japan focus on patients with depression. However, the illnesses that lead to leave of absence are diverse. Hence, an approach based on ACT, which has no diagnostic specificity, may be beneficial and efficient for RTW support (Hagberg et al., 2023). Meanwhile, this study included participants with various conditions such as adjustment disorders, depression, and developmental disorders, and it will be necessary to continuously consider cross-diagnostic RTW support in Japan as well.

Nevertheless, this study has several limitations. First, the absence of a control group makes the findings inconclusive. Second, as participants could receive pharmacotherapy or other psychological interventions, the potential overlap in these interventions’ effects could not be excluded. Third, we assessed symptoms using self-report questionnaires rather than objective measurement methods, such as structured clinical interviews. Given the nature of functional assertiveness, not only the speaker’s self-assessment but also whether the expression is actually acceptable to the listener should be evaluated. Fourth, as the AAQ-II has construct issues, caution should be taken in selecting the appropriate scale, such as considering the Comprehensive Assessment of Acceptance and Commitment Therapy or AAQ-III, as suggested by Ong et al. (2020). Fifth, this study assessed symptoms only pre- and post-treatment but did not evaluate long-term outcomes after RTW or whether the patients actually RTW. Therefore, the long-term efficacy of the program was not tested. Sixth, we were unable to continue recording the attendance of participants due to a careless mistake by a staff member; therefore, we were unable to examine the relationship between attendance and the program’s effectiveness. Finally, future research should build on our findings and examine the relationship between the components of ACT and functional self-assertion in detail. In particular, in this study, even though some elements of mindfulness did not change, functional assertion and sense of difficulty in RTW did change, so it will be necessary to examine the relationship between these in detail. Additionally, researchers should consider individual differences such as gender, age, and work environment. It is also desirable to use high-precision research designs, such as research designs using Treatment as Usual (TAU) groups that take into account confounding factors. By doing so, future research can validate the appropriateness of incorporating ACT into functional assertiveness programs and examine the long-term effects on employees returning to work.

## Data Availability

The raw data supporting the conclusions of this article will be made available by the authors, without undue reservation.
